# Efficacy, pharmacokinetics, and safety of adalimumab in pediatric patients with juvenile idiopathic arthritis in Japan

**DOI:** 10.1007/s10067-012-2082-5

**Published:** 2012-10-02

**Authors:** Tomoyuki Imagawa, Syuji Takei, Hiroaki Umebayashi, Kenichi Yamaguchi, Yasuhiko Itoh, Toshinao Kawai, Naomi Iwata, Takuji Murata, Ikuo Okafuji, Mari Miyoshi, Yasuhiro Onoe, Yoshifumi Kawano, Noriko Kinjo, Masaaki Mori, Neelufar Mozaffarian, Hartmut Kupper, Sourav Santra, Gina Patel, Shinichi Kawai, Shumpei Yokota

**Affiliations:** 1 Yokohama City University, Yokohama, Japan; 2 Kagoshima University, Kagoshima, Japan; 3 Miyagi Children’s Hospital, Miyagi, Japan; 4 St. Luke’s International Hospital, Tokyo, Japan; 5 Nippon Medical School Hospital, Tokyo, Japan; 6 National Center for Child Health and Development, Tokyo, Japan; 7 Aichi Children’s Health and Medical Center, Aichi, Japan; 8 Osaka Medical College Hospital, Takatsuki City, Japan; 9 Kobe City Medical Center General Hospital, Hyogo, Japan; 10 Hyogo Prefectural Kobe Children’s Hospital, Hyogo, Japan; 11 National Hospital Organization Kokura Medical Center, Kitakyushu, Japan; 12 Kagoshima University Medical and Dental Hospital, Kagoshima, Japan; 13 Hospital, University of the Ryukyus, Okinawa, Japan; 14 Abbott Laboratories, Abbott Park, IL USA; 15 Abbott GmbH & Co. KG, Ludwigshafen, Germany; 16 GlaxoSmithKline Pharmaceuticals, Limited, Bangalore, India; 17 Toho University School of Medicine, Tokyo, Japan; 18 Department of Pediatrics, Yokohama City University School of Medicine, 3-9 Fukuura, Kanazawa-ku, Yokohama, Kanagawa 236-0004 Japan

**Keywords:** Adalimumab, Juvenile idiopathic arthritis, Methotrexate, Pharmacokinetics

## Abstract

The objective of this study was to evaluate the efficacy, pharmacokinetics, and safety of adalimumab in patients with polyarticular juvenile idiopathic arthritis (JIA) in Japan. Patients aged 4 to 17 years were enrolled in a single-arm, open-label, multicentre study of adalimumab. Patients weighing <30 kg received 20 mg every other week (eow), and those ≥30 kg received 40 mg eow. Concomitant methotrexate (MTX) was allowed (≤10 mg/m^2^ per week). The primary efficacy outcome was the percent of patients with American College of Rheumatology Pediatric 30 response (ACR Pedi 30) at week 16. JIA core variables, serum adalimumab concentrations, and anti-adalimumab antibodies (AAAs) were analysed. Patients were monitored for adverse events (AEs). Twenty-five patients (20 with concomitant MTX at baseline and 5 without) were enrolled: 24 patients completed 16 weeks of therapy and 22 patients completed 60 weeks. At week 16, 90 % of patients with MTX and 100 % without MTX achieved ACR Pedi 30; response rates were maintained through week 60 in 94 and 80 % of patients, respectively. Each JIA core variable improved over time. Six patients became AAA positive (two each at weeks 8, 16, and 60), some of which were transient. All six AAA-positive patients achieved ACR Pedi 30 at week 16, and four maintained that response at week 60. Six patients (all with MTX) experienced nine serious AEs (JIA, pyrexia, arthralgia, pneumonia, hepatitis B infection, pharyngitis, dehydration, pharyngeal pain, and pneumonia). In pediatric patients with polyarticular JIA in Japan, adalimumab was safe and effective for reducing disease activity for up to 60 weeks.

## Introduction

Juvenile idiopathic arthritis (JIA) is the most common rheumatic disease in children <16 years of age [1] and often causes significant pain, functional impairment, and diminished health-related quality of life [2–5]. The incidence of JIA in Japan is estimated to be 10 to 15 per 100,000 children [6]. The overall incidence in developed countries is estimated to range from 16 to 150 per 100,000 children [1]. Of the JIA subtypes, the incidence of polyarticular JIA, which affects five or more joints, has been reported to be relatively high among children in Japan, whereas pauciarticular JIA, which affects four or less joints, is more common in Europe and the USA [6].

The Pediatric Standing Committee of the Japan College of Rheumatology and the Pediatric Rheumatology Association of Japan collaborated to produce guidelines for the diagnosis and treatment of JIA in Japan, with an emphasis on the involvement of pediatric rheumatologists, early intervention, and individualised therapy [6]. For articular JIA, nonsteroidal anti-inflammatory drugs (NSAIDs) are the first-line therapy of choice, followed by disease-modifying antirheumatic drugs (DMARDs) such as methotrexate (MTX) and oral corticosteroids [6]. Patients with JIA often have persistent disease. Consequently, the use of these agents may be limited by long-term toxicity, including potential effects on growth, partial/inadequate responses, and inability to maintain disease remission [7–9]. Short-term toxicities and drug intolerance are also an issue in this population. Some patients with JIA may benefit from treatment with biologic agents. Further guidance on this topic was recently published by the American College of Rheumatology (ACR) and is under development elsewhere [10, 11]. Availability of biologic therapies, including the anti-tumour necrosis factor (anti-TNF) agents adalimumab, etanercept, and infliximab, the T-cell inhibitor abatacept, and the humanised anti-human interleukin 6 receptor monoclonal antibody tocilizumab, represents a major advancement in the treatment of rheumatic diseases. Of these agents, etanercept, tocilizumab, and adalimumab are approved for JIA in Japan [11–13].

Adalimumab is a fully human monoclonal antibody specific to human TNF-alpha that is approved in the USA (February 2008) and Europe (September 2008) for the treatment of polyarticular JIA and was recently approved for JIA in Japan [13–15]. TNF, involved in normal inflammatory and immune responses, is also elevated in the synovial fluid of JIA patients. Adalimumab binds specifically to TNF-alpha and blocks its interaction with cell surface TNF receptors. The relationship between the pharmacodynamic action of adalimumab and the mechanism(s) by which adalimumab exerts its clinical effects is unknown but has been hypothesized to involve significant inhibition of the pathologic inflammatory process. Adalimumab has been shown to be safe and effective in patients with JIA when dosed every other week (eow) for up to 3 years in Western populations [16]. Further, these patients maintained clinical responses and improvements in disease activity for up to 6 years, with no deaths or malignancies reported [17]. The primary objective of this study was to evaluate the efficacy, pharmacokinetics, and safety of adalimumab therapy in pediatric patients with polyarticular JIA in Japan.

## Materials and methods

### Patients

#### Main inclusion criteria

Pediatric patients aged 4 to 17 years with a diagnosis of polyarticular JIA by ACR criteria at screening, and active disease defined as five or more swollen joints (not due to deformity) and three or more joints with limitation of passive motion due to pain and/or pain by pressure (tenderness), were eligible. Patients could have had systemic, polyarticular, or pauciarticular JIA at disease onset but were required to be free of any systemic JIA manifestations for at least 12 weeks before screening. All patients were to have had inadequate clinical response (or intolerance) to NSAIDs or MTX (8–10 mg per 1 m^2^ of body surface area per week for 12 weeks or more), or other conventional therapies for JIA such as corticosteroids. Negative urine pregnancy tests at screening and baseline were required for all postpubertal females; use of adequate contraception during the study and for 150 days after last injection was required for all sexually active patients.

#### Main exclusion criteria

Patients with a history of or comorbid inflammatory joint disease other than JIA, including psoriatic arthritis, arthritis related to enthesopathy, and systematic lupus erythematosus, were excluded. Patients classified as functional class IV by ACR criteria, prior treatment with alkylating agents or biologic therapies for JIA, joint surgery within 8 weeks prior to screening, or articular corticosteroids or sodium hyaluronate within 28 days prior to baseline were also excluded. Other exclusion criteria included the following: significant cardiac disease; abnormal laboratory values; positive serology for anti-human immunodeficiency virus antibody, hepatitis B surface antigen, or hepatitis C antibody; history of a central nervous system (CNS) neoplasm; active CNS infection, demyelinating disease, degenerative neurological disease, or any progressive CNS disease; ongoing chronic or active infection or any major episode of infection requiring hospitalisation or treatment with intravenous antibiotics within 28 days or oral antibiotics within 14 days prior to screening; history of Listeria infection; tuberculosis confirmed by chest X-ray or chest computed tomography at screening or confirmed history of tuberculosis; history of cancer, lymphoma, leukemia, or lymphoproliferative disease other than a successfully treated nonmetastatic cutaneous squamous cell or basal cell carcinoma and/or localised carcinoma in situ of the cervix; and vaccination with a live vaccine within 12 weeks of study drug administration through 70 days of the last dose.

#### Approved/prohibited medications

MTX was either to be discontinued at least 14 days prior to baseline or, for patients receiving a stable dose (≤10 mg/m^2^ per week) for ≥12 weeks prior to baseline, could be continued at the stable dose until week 16. After week 16, dose reduction or discontinuation of MTX was permitted based on investigator’s judgement. If MTX was reduced or interrupted, it could subsequently be increased or restarted as long as the dose was ≤10 mg/m^2^ per week.

DMARDs other than MTX were to be discontinued at least 28 days before baseline. NSAIDs or low-dose corticosteroids (≤0.2 mg/kg of prednisone per day, up to a maximum of 10 mg/day, including subcutaneous or intramuscular injection) were permitted at stable doses. Pain medications were not to be taken within 12 h prior to the study visit joint evaluation. Intraarticular injection of corticosteroids or sodium hyaluronate was prohibited until week 16, after which time, one intraarticular injection of corticosteroid or sodium hyaluronate was permitted; however, the joint was considered nonevaluable for 12 weeks after injection. Use of immunosuppressants or leukocytapheresis, alkylating agents such as cyclophosphamide, and analgesic opium alkaloid/synthetic narcotics was prohibited.

### Study design and ethics

This single-arm, open-label, multicentre study evaluating the efficacy, pharmacokinetics, and safety of fixed dose adalimumab in pediatric patients with polyarticular JIA was conducted at 14 sites in Japan from May 2008 to August 2010 (NCT00690573). After a 2- to 4-week screening period, patients received subcutaneous adalimumab injections eow. Based on pharmacokinetic simulations performed using body surface area-dependent dosing data from a Western (USA and Europe) clinical JIA study (NCT00048542) [16], a fixed dose dosing regimen was established in which two different doses of adalimumab were administered based on body weight. Patients weighing <30 kg received adalimumab 20 mg eow and patients weighing ≥30 kg received adalimumab 40 mg eow. Adalimumab dosage was re-evaluated based on actual body weight at week 16 and every 12 weeks after week 24 through week 60.

The study was conducted in accordance with the Declaration of Helsinki. In accordance with harmonised good clinical practices, institutional review boards approved the study protocol and the informed consent form. The patient or parent or legal guardian of the patient reviewed and voluntarily signed the informed consent form prior to involvement in any study-related procedures.

#### Clinical assessments

Clinical measures were assessed at weeks 2, 4, 8, 16, and 24 and every 12 weeks after week 24. The following JIA core criteria were used to calculate ACR Pedi scores: Physician’s Global Assessment of disease activity (PhGA), Patient’s Global Assessment of well-being (PaGA), active joint count in 73 joints (AJC73), limitation of motion in 69 joints (LOM69), Childhood Health Assessment Questionnaire (CHAQ) to assess physical function, and C-reactive protein (CRP) concentration. ACR Pediatric 30 (ACR Pedi 30) response was defined as ≥30 % improvement in at least three of six JIA core criteria and ≥30 % worsening in not more than one of six JIA core criteria. ACR Pedi 50/70/90 responses were defined similarly according to the respective level of response for at least three of the six JIA core criteria and ≥30 % worsening in not more than one of six JIA core criteria.

#### Pharmacokinetics and immunogenicity

Blood samples for analysis of serum adalimumab concentrations and anti-adalimumab antibodies (AAAs) were collected at baseline and weeks 2, 4, 8, 16, 24, 36, 48, and 60. A validated enzyme-linked immunoadsorbent assay method based on a double-antigen technique was used to measure adalimumab and AAA concentrations. The lower limit of quantitation (LLOQ) for adalimumab was 3.13 ng/mL in diluted (1:10) serum or 31.3 ng/mL in undiluted serum. The LLOQ for AAAs was 1.0 ng/mL in diluted serum or 10 ng/mL in undiluted serum. Samples were considered AAA-positive if the measured AAA concentration was >20 ng/mL in undiluted serum, and the sample was collected within 30 days after an adalimumab dose. Serum samples with adalimumab concentrations >2 μg/mL were not analysed for AAAs.

#### Safety

The investigator monitored each patient for clinical and laboratory evidence of adverse events (AEs) on a routine basis throughout the study. AEs were also assessed at a follow-up visit 28 days after study completion and 70 days after the last dose of study drug (by site visit or telephone). Duration of exposure was calculated as the number of days between the first dose of study drug and the last dose plus 14 days.

### Statistical analysis

As per the prespecified analysis plan, efficacy analyses were conducted on the full analysis set, which was defined as all patients who received at least one dose of study drug. The primary efficacy endpoint, ACR Pedi 30 response rate at week 16, was analysed with non-responder imputation (NRI), whereby patients with missing data at week 16 or who failed to meet ACR Pedi 30 response at week 16 were considered non-responders. Secondary endpoints, including longitudinal data for ACR Pedi 30/50/70/90 response rates and the JIA core variables from week 16 through week 60, were analysed using observed cases (i.e., only patients who were in the study at the time point being evaluated were included). Serum adalimumab and AAA concentrations were summarised descriptively. The numbers and percentages of patients who experienced AEs with an onset on or after the first adalimumab injection up to 70 days (five half-lives) after the last injection were summarised for the safety analysis set, which was identical to the full analysis set.

## Results

### Patient disposition and duration of treatment

A total of 25 children with JIA were treated with adalimumab, of which 20 were receiving concomitant MTX at baseline. Twenty-four patients completed 24 weeks of therapy, including the primary endpoint assessment at week 16, and 22 patients completed up to 60 weeks. The three patients who discontinued the study, all due to lack of efficacy, were in the concomitant MTX group. The average duration of adalimumab treatment through week 60 was 396 days (390 days for patients receiving concomitant MTX at baseline and 421 days for patients without MTX).

### Baseline demographics and clinical characteristics

At baseline, the mean age was approximately 13 years, 80 % of the patients were female, and 32 % weighed <30 kg (Table 1). For patients who received MTX (*N* = 20) and those who did not (*N* = 5), respectively, the duration of JIA was 4.8 and 4.2 years, 70 and 60 % were rheumatoid factor positive (RF positive), and 55 and 60 % had abnormal (>3 mg/dL) CRP concentrations. All patients had polyarticular JIA at disease onset, except for one patient (not receiving MTX) who had pauciarticular JIA at disease onset.

**Table 1:** Demographics and clinical characteristics at baseline

	With MTX	Without MTX	All adalimumab
	(*N* = 20)	(*N* = 5)	(*N* = 25)
Age			
Mean (SD), years	13.2 (3.22)	12.6 (4.39)	13.0 (3.38)
4–12 years, *n* (%)	9 (45)	3 (60)	12 (48)
13–17 years, *n* (%)	11 (55)	2 (40)	13 (52)
Female, *n* (%)	15 (75)	5 (100)	20 (80)
Body weight			
Mean (SD), kg	40.5 (11.28)	35.3 (16.64)	39.5 (12.31)
≥30 kg, *n* (%)	14 (70)	3 (60)	17 (68)
Duration of JIA, mean (SD), years	4.8 (3.97)	4.2 (2.75)	4.7 (3.72)
RF positive, *n* (%)	14 (70)	3 (60)	17 (68)
Anti-CCP antibody, mean (SD), U/mL	105.5 (135.67)	8.5 (15.50)	86.1 (127.19)
LOM69, mean (SD)	8.6 (5.65)	5.8 (2.05)	8.0 (5.22)
AJC73, mean (SD)	12.0 (6.10)	13.6 (9.32)	12.3 (6.66)
CRP concentration			
Abnormal (>0.3 mg/dL), *n* (%)	11 (55)	3 (60)	14 (56)
Mean (SD), mg/dL	1.0 (1.32)	3.6 (3.86)	1.5 (2.22)
CHAQ (0–3), mean (SD)	0.8 (0.79)	0.7 (1.11)	0.8 (0.84)
PhGA (0–100 mm), mean (SD)	56.5 (18.49)	58.6 (25.83)	56.9 (19.56)
PaGA (0–100 mm), mean (SD)	44.6 (24.84)	48.6 (34.20)	45.4 (26.19)

*AJC* active joint count, *CCP* cyclic citrullinated protein, *CHAQ* Childhood Health Assessment Questionnaire, *CRP* C-reactive protein, *JIA* juvenile idiopathic arthritis, *LOM* limitation of motion, *MTX* methotrexate, *PaGA* Patient’s Global Assessment, *PhGA* Physician’s Global Assessment, *RF* rheumatoid factor, *SD* standard deviation

Baseline PhGA, PaGA, and CHAQ scores were similar between groups. Baseline anti-cyclic citrullinated protein (anti-CCP) antibody concentrations were considerably greater in patients who were receiving MTX compared with those not receiving MTX. All patients had received DMARDs previously. At baseline, all patients were receiving concomitant NSAIDs, and most patients were also receiving concomitant systemic corticosteroids (70 % in the MTX group and 80 % in the non-MTX group).

### Efficacy

#### Primary endpoint: ACR Pedi 30 response

**Fig. 1:**
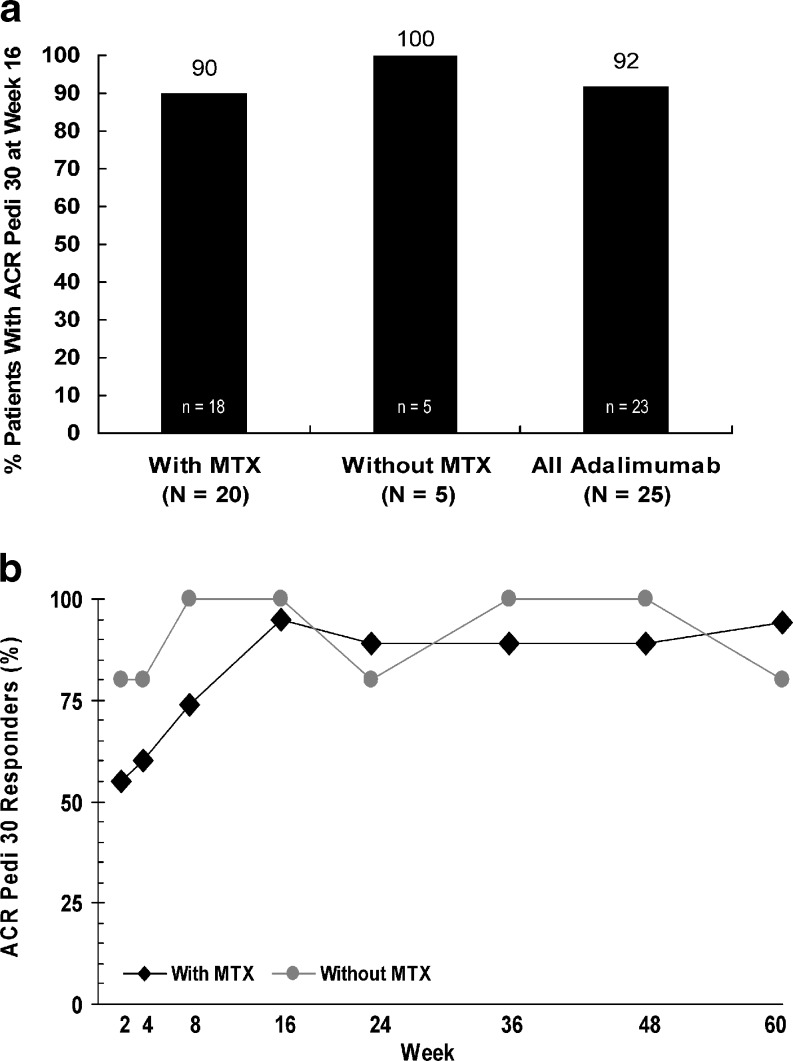
ACR Pedi 30 response rates. **a** Primary efficacy outcome: ACR Pedi 30 response rates at week 16 of adalimumab therapy (NRI). **b** ACR Pedi 30 response rates over time with adalimumab therapy (as observed) (*black diamond* with MTX, *grey circle* without MTX)

Overall, 92 % of patients achieved ACR Pedi 30 at week 16 of adalimumab therapy (90 % in the MTX group and 100 % in the non-MTX group) (Fig. 1a). ACR Pedi 30 response at week 60 was observed for 94 % of patients with concomitant MTX and 80 % of patients without concomitant MTX (Fig. 1b).

#### ACR Pedi 50/70/90 responses

ACR Pedi 50/70 response rates at week 16 of adalimumab therapy were generally consistent with those at week 60, with approximately 90 and 75 % of patients achieving these levels of response. Overall, ACR Pedi 90 response rates increased from ≤20 % at week 16 to 50 % at week 60.

#### JIA core variables over time

Adalimumab therapy was associated with improvements in each of the six JIA core variables over time (Table 2). Mean decreases in disease activity generally started as early as week 2 (data not shown) and remained consistent with improvements observed through week 60.

**Table 2:** JIA core variables at weeks 16 and 60 of adalimumab therapy (as observed)

	Adalimumab with MTX			Adalimumab without MTX		
	Mean baseline value	Mean visit value	Mean % change	Mean baseline value	Mean visit value	Mean % change
Week 16						
PhGA (mm)	55.8	20.6	−64.8	58.6	10.6	−83.8
PaGA (mm)	44.7	22.1	−50.5	48.6	15.4	−74.5
AJC73	11.0	3.8	−59.9	13.6	3.2	−80.3
LOM69	7.5	3.7	−38.3	5.8	1.6	−76.7
CHAQ	0.7	0.4	−32.8	0.7	0.4	−35.7
CRP (mg/dL)	0.9	0.3	−23.8	3.6	1.7	−65.1
Week 60						
PhGA (mm)	55.0	16.7	−74.0	58.6	12.8	−81.5
PaGA (mm)	42.9	16.6	−51.7	48.6	27.0	−48.6
AJC73	11.1	1.9	−81.5	13.6	3.0	−79.8
LOM69	7.6	1.5	−78.7	5.8	2.4	−72.2
CHAQ	0.7	0.2	−46.9	0.7	0.5	−41.0
CRP (mg/dL)	1.0	0.4	−46.6	3.6	1.4	−4.0

*AJC* active joint count, *CHAQ* Childhood Health Assessment Questionnaire, *CRP* C-reactive protein, *LOM* limitation of motion, *MTX* methotrexate, *PaGA* Patient’s Global Assessment, *PhGA*, Physician’s Global Assessment

#### Pharmacokinetics and immunogenicity

**Fig. 2:**
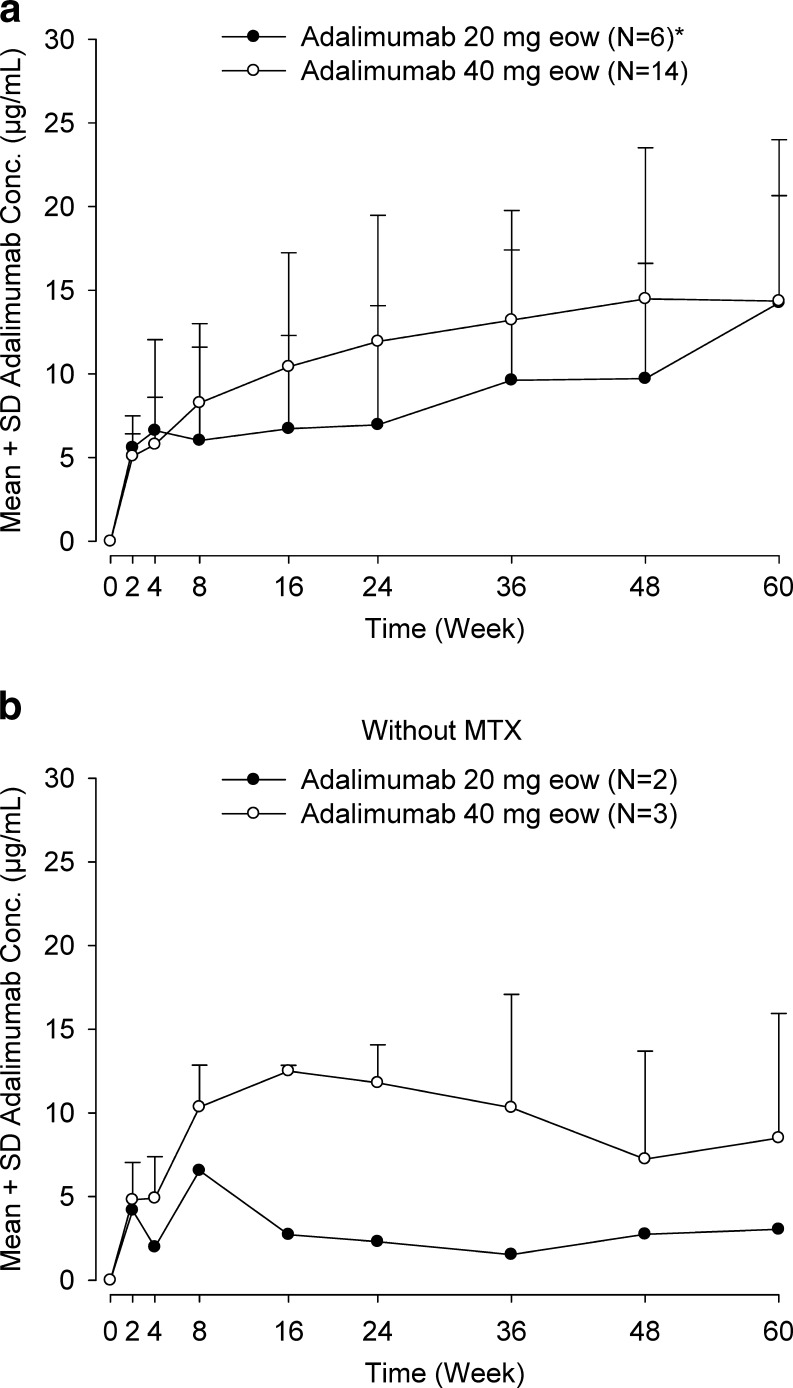
Serum adalimumab concentrations. **a** Mean concentration for all patients (*N* = 20) with concomitant MTX by dosage of adalimumab (*black circle* 20 mg eow, *open circle* 40 mg eow). **b** Mean concentration in patients without concomitant MTX (*N* = 5) by dosage of adalimumab (*black circle* 20 mg eow, *open circle* 40 mg eow). *Asterisk* Adalimumab dosages were increased from 20 to 40 mg eow at week 16 for two patients, at week 36 for one patient, and at week 48 for one patient owing to body weight increases (<30 kg at baseline to ≥30 kg at week 16), as per protocol

Mean adalimumab concentrations for patients who received adalimumab 40 mg eow plus MTX were 10.4 μg/mL (range, 0–19.4 μg/mL; *N* = 14) at week 16 and 14.4 μg/mL (range, 0–21.6 μg/mL; *N* = 14) at week 60 (Fig. 2a). Mean adalimumab concentrations for patients who received adalimumab 20 mg eow plus MTX were 6.73 μg/mL (range, 0–13.2 μg/mL; *N* = 6) at week 16 and 14.3 μg/mL (range, 0–24.6 μg/mL; *N* = 6) at week 60; two of the six patients initially receiving the 20-mg dosage increased to 40 mg by week 60 due to changes in body weight. For patients receiving adalimumab without MTX, mean adalimumab concentrations were consistent with mean concentrations in patients receiving adalimumab plus MTX (Fig. 2b).

At week 24 of adalimumab therapy, 16 % (4 of 25) patients had at least one AAA-positive serum sample (3 of 20 with MTX [15 %] and 1 of 5 without MTX [20 %]). At week 60, 15 % (3 of 20) of patients receiving adalimumab plus MTX and 60 % (3 of 5) of patients receiving adalimumab without MTX had detectable AAAs (6 of 25 patients [24 %] overall). Two patients first had detectable AAAs at week 8, two at week 16, and two at week 60. Of these six patients, three patients were receiving the 20-mg dosage (one with MTX and two without MTX) and three were receiving the 40 mg dosage (two with MTX and one without MTX).

Trough concentrations of adalimumab <2 g/mL were transient for three of the four patients in whom AAAs were detected prior to week 60. For two of these four patients (one with MTX and one without MTX), the lack of measurable serum adalimumab at a given study visit was followed by detectable adalimumab concentrations that increased at later time points through week 60. Another patient (with MTX) had AAAs detected at week 8 but had measurable adalimumab concentrations at weeks 36 and 48 following dosage increase to 40 mg eow at week 16; the patient’s adalimumab concentration was again undetectable at week 60. The remaining patient (with MTX) had undetectable adalimumab concentrations from week 8 to week 60. All six patients with AAA at any time point achieved ACR Pedi 30 response at week 16 and completed the study; four of the six patients exhibited ACR Pedi 30 response at week 60.

### Safety

Through week 60, all patients had experienced at least one AE (Table 3). Six patients (all with concurrent MTX) experienced nine serious AEs as follows: JIA, pyrexia, and arthralgia; pneumonia; hepatitis B infection; pharyngitis; dehydration, pharyngeal pain, and pyrexia. Consistent with study exclusion criteria, the patient with hepatitis B infection had been negative for hepatitis Bs antigen and antibodies against HBs and HBc at the start of the trial; no risk factors for transmission (i.e., travel to endemic areas, injection drug use, or other exposure) were reported, and the patient discontinued the study because of this event. Of the six patients with serious AEs, four occurred in AAA-negative patients and two occurred in AAA-positive patients (one with serious AE of JIA; one with serious AEs of dehydration, pharyngeal pain, and pneumonia).

**Table 3:** Patients with treatment-emergent adverse events

Adverse Event	Adalimumab with MTX	Adalimumab without MTX	All adalimumab
	(*N* = 20)	(*N* = 5)	(*N* = 25)
	*n* (%)	*n* (%)	*n* (%)
Any AE	20 (100)	5 (100)	25 (100)
Any severe AE	0	0	0
Any AE leading to discontinuation of study drug	1 (5)	0	1 (4)
Any serious AE	6 (30)	0	6 (24)
Any infectious AE	16 (80)	5 (100)	21 (84)
Any serious infectious AE	3 (15)	0	3 (12)
Injection-site reaction	6 (30)	0	6 (24)
Hepatic-related AE	2 (10)	1 (20)	3 (12)

*AE* adverse event, *MTX* methotrexate

#### AEs of special interest

Infections occurred in 80 % of patients receiving MTX and 100 % of patients not receiving MTX, of which three in the MTX group were serious (acute pharyngitis, pneumonia, and hepatitis B infection). Six patients had an injection-site reaction (erythema, swelling, warmth, all in the MTX group) and three had a hepatic event (two in the MTX group, hepatic function abnormal and blood alkaline phosphatase increased; one in the non-MTX group, hepatic function abnormal). No deaths, malignancies, opportunistic infections, congestive heart failure, demyelinating disease, allergic reactions, lupus-like syndrome, or blood dyscrasias were reported in any patient through week 60 of the study.

## Discussion

Polyarticular JIA with RF positivity, which represents approximately 5 % of patients with JIA, tends to be more aggressive than RF-negative polyarticular JIA and is associated with early joint destruction and disability [18, 19]. In addition, RF-positive JIA patients have been found to be less likely to achieve a state of disease inactivity and spend more time in an active disease state compared with RF-negative patients [20]. In the present study, 68 % of patients were RF positive, thus representing a population of patients with potentially severe and difficult-to-treat JIA who are candidates for intensive therapy. Results of this study demonstrate the efficacy of adalimumab in reducing disease activity and maintaining response through 60 weeks of therapy in pediatric patients with polyarticular JIA in Japan. Because the majority of patients in the study were receiving concomitant MTX, firm conclusions regarding the efficacy of adalimumab monotherapy vs. combination therapy are limited; however, adalimumab-treated patients receiving combination therapy with MTX in the Western JIA study had numerically higher ACR Pedi 30, 50, 70, and 90 responses compared with those not receiving MTX [16].

AAAs may develop in some patients during adalimumab therapy. In pediatric patients with JIA in Japan, an AAA-positive result was not associated with study discontinuation or lack of efficacy; all six patients with AAAs completed the study, and four achieved at least ACR Pedi 30 at week 60. AAA positive rates in pediatric patients on concomitant MTX were numerically different, with a higher rate in Japanese vs. Western patients, 15 % (3/20) vs. 5.9 % (5/85), respectively [16]. JIA rates appear consistent with rates observed in Japanese and Western adult RA populations; however, limited pediatric data preclude any correlation with adult RA. In this study, the overall sample size, particularly of the non-MTX group, was too small to make any meaningful comparison of AAA rates to the Western JIA study population.

The similarity of the inclusion criteria between the present study conducted in Japan and the Western JIA study [16] allows for comparison of the results. Disease duration (approximately 4 years) and baseline JIA core criteria generally were similar between patients in both studies; however, a greater percentage of patients in Japan were RF positive at baseline and had received prior DMARD therapy. Notably, ACR Pedi 30 rates at week 16 for patients receiving adalimumab plus MTX were comparable between both studies, with >90 % of patients in each study achieving this efficacy endpoint.

In adult RA, the overall safety profile of adalimumab in Japanese studies is consistent with that observed in Western studies [21–23]. Similarly, the safety profile of adalimumab in patients with polyarticular JIA in Japan was as expected, based on the safety profile observed in the Western JIA study [16], and no new AEs of interest were observed. Although infections were the most common AE of interest in the present study, no opportunistic infections or tuberculosis were reported through 60 weeks of adalimumab therapy. One event of hepatitis B infection was reported in the present study, and hepatitis B evaluation is recommended for patients receiving biologic therapy.

In addition to adalimumab, two other biologic agents (etanercept and tocilizumab) are approved for the treatment of JIA in Japan. Although head-to-head trials have not been conducted, clinical efficacy as measured by ACR Pedi 30 response rates appears to be relatively similar among these drugs. In a study of DMARD-refractory polyarticular JIA in Japan, etanercept treatment resulted in an ACR Pedi 30 response rate of 91 % at week 12 of the primary study and 94 % at week 96 of the open-label extension [12]. For the anti-interleukin-6 receptor antibody tocilizumab, approved for DMARD-refractory polyarticular JIA and systemic JIA [11], the ACR Pedi 30 response rate was 95 % at week 12 and 100 % at week 48 [24].

Limitations of this study include the open-label study design and small sample size, especially for patients not receiving MTX. In addition, the effect of adalimumab on radiographic progression could not be assessed because X-ray data were not collected. Although the absence of valid and reliable radiographic scoring systems in children with JIA is the primary reason radiographic outcomes have not been included in pivotal JIA trials, measures such as the adapted Sharp-van der Heijde score have been validated and are recommended for use in future pediatric JIA studies [25, 26].

The efficacy, safety, and tolerability of adalimumab were demonstrated and maintained through 60 weeks of therapy in patients with JIA in Japan. The results of the present study were similar to those from a JIA study conducted in the USA and Europe [16]. AAA-positive rates were numerically greater in JIA patients in Japan; however, the development of these antibodies was not associated with study discontinuation or with lack of efficacy. In conclusion, fixed-dose adalimumab (20 or 40 mg eow) was effective in reducing disease activity in pediatric patients with polyarticular JIA in Japan.
